# Impact of Combined Additional Resections on the Surgical Outcomes of Robot‐Assisted Resection of Thymic Epithelial Tumors

**DOI:** 10.1002/jso.70162

**Published:** 2025-12-18

**Authors:** Benedikt Niedermaier, Nabil Khan, Florian Eichhorn, Maria Zehentmeier, Heidrun Grosch, Raffaella Griffo, Alessio Campisi, Antonia Margineanu, Michael Allgäuer, Petros Christopoulos, Michael Thomas, Hauke Winter, Martin E. Eichhorn

**Affiliations:** ^1^ Department of Thoracic Surgery Thoraxklinik at the University of Heidelberg Heidelberg Germany; ^2^ Translational Lung Research Center Heidelberg (TLRC‐H) Member of the German Center for Lung Research (DZL) Heidelberg Germany; ^3^ Division of Systems Biology of Signal Transduction, German Cancer Research Center (DKFZ) DKFZ‐ZMBH Alliance Heidelberg Germany; ^4^ Department of Thoracic Oncology, Thoraxklinik, Heidelberg University Hospital and National Center for Tumor Diseases (NCT) NCT Heidelberg, a partnership between DKFZ and Heidelberg University Hospital Heidelberg Germany; ^5^ Thoracic Surgery, Department of Surgery, P. Pederzoli Hospital Peschiera Del Garda Verona Italy; ^6^ Department of Pathology University Hospital Heidelberg Heidelberg Germany

**Keywords:** advanced stage, case series, perioperative outcomes, robot‐assisted, thymoma

## Abstract

**Background and Objectives:**

Robot‐assisted thoracoscopy (RATS) is rapidly emerging as the preferred approach for the resection of thymic epithelial tumors (TET). Current challenges include the role of RATS in locally advanced disease and combined additional resections.

**Methods:**

This single‐center study included all consecutive robot‐assisted surgeries for TET performed between 2018 and 2024. We report perioperative outcomes and findings from a large center for robotic surgery center.

**Results:**

One hundred and forty‐three patients underwent RATS for the resection of histologically confirmed TET, including 130 (91%) patients with thymoma and 13 (9%) patients with thymic carcinoma. The median tumor size was 54 mm (35.5–75) and most patients presented in a localized stage of disease, with 120 patients (83.9%) in TNM stage I (TNM 8th edition). The conversion rate to open surgery was 4.2% and R0 resection was achieved in 134 (93.7%) patients. Combined extended resections that included lung, pericardium or great vessels were performed in 44 (30.8%) patients and were the only independent predictor of postoperative complications in a multivariable logistic regression model (OR 2.87; *p* = 0.03).

**Conclusions:**

Robot‐assisted surgery is feasible and without unexpected safety concerns for TET. Combined extended resections, often necessary for locally advanced disease, are a significant predictor of postoperative complications.

## 1 Introduction

Thymic epithelial tumors (TET) are rare malignant tumors of the anterior mediastinum, which include thymomas and thymic carcinomas with a total annual incidence of 2–3 cases per million [1]. The WHO classification of TET classifies thymomas based on morphologic and molecular characteristics and distinguishes between type A, AB, B1, B2, and B3; as well as metaplastic thymomas and micronodular thymomas with lymphoid stroma (MTWLS) [2, 3]. Thymic carcinomas can occur as various histopathologic subtypes, such as squamous cell carcinoma or basaloid carcinoma and many others [2]. For resectable tumors, such as Stage I/II Masaoka‐Koga tumors and some stage III tumors, primary resection is the recommended treatment [4]. Complete resection with negative margins has been established as the most important predictor of survival and the gold‐standard has traditionally been median sternotomy or thoracotomy [4, 5]. In recent years, robot‐assisted thoracoscopy (RATS) has rapidly emerged as an alternative to open procedures in mediastinal surgery, and is now becoming the standard of care in many centers around the world [6, 7, 8]. Advantages of RATS include less surgical trauma, fewer complications, shorter postoperative stay, better cosmetic results and faster recovery of lung function [9, 10, 11]. However, challenges remain. While RATS is well established for minimally‐invasive resection of small thymic tumors, there are currently limitations in the use of RATS including uncertainties related to locally advanced disease, combined additional resections, neoadjuvant treatment strategies, maximum feasible tumor size and management and prediction of complications. Here we present our clinical experience and findings from a large cohort of patients with TET who underwent curative‐intent resection by RATS.

## 2 Methods

### 2.1 Study Population

**Figure 1:**
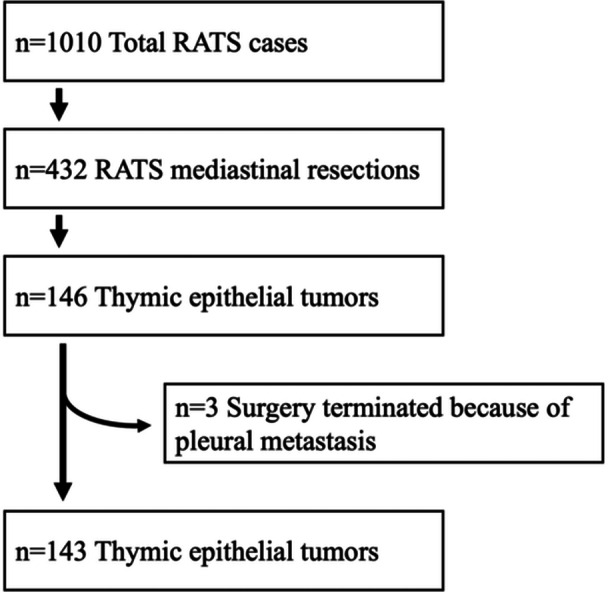
Flow chart for the selection of patients.

This retrospective study was conducted on the basis of a prospective database including all robot‐assisted surgeries, and was approved by the local ethics committee (S‐089/2018). The study adhered to the declaration of Helsinki on ethical principles in medical research. Informed consent was obtained from all patients. Among 146 consecutive patients undergoing RATS for histologically confirmed thymic epithelial tumors, three patients were excluded because surgery was aborted and tumor resection was not performed after extensive pleural carcinomatosis was detected intraoperatively, resulting in a final study population of 143 patients (Figure 1). Tumor stage was reported according to the Masaoka‐Koga classification and the 8th edition of the IASLC/ITMIG TET Staging Project TNM classification [12]. Tumor size was reported as the maximum tumor diameter measured at final pathology. Complications were reported according to the Clavien‐Dindo classification [13].

### 2.2 Perioperative Management

Our routine diagnostic approach included preoperative CT‐imaging and pulmonary function tests. If infiltration into neighboring vessels or other important anatomical structures could not be ruled out, an MRI was performed for further evaluation [14]. The individual cases were discussed by an interdisciplinary board and the decision in favor of RATS was made if resection appeared technically feasible and oncologically reasonable. All operations were conducted using the DaVinci X System (Intuitive Surgical, Sunnyvale, California, USA) with a dual console as previously described [9, 15]. Postoperative follow‐up visits were conducted every 3 months for the first 2 years after surgery, every 6 months until the fifth year, and annually thereafter according to current guidelines [4].

### 2.3 Statistical Analysis

Statistical analyses were performed using SPSS Statistics (IBM Corp., v29) and Prism (Graphpad Software Inc., v8). Categorical variables were summarized as counts and percentages. The distribution of continuous variables was tested for normality using the Shapiro–Wilk test and visualized with histograms. As none of the continuous variables met the assumption of normality, they were summarized using the median and interquartile range (IQR). To examine risk factors for postoperative complications, univariable and multivariable logistic regression analysis was performed. A stepwise backward elimination approach was used to select the best‐fitting subset of variables for the final multivariable model. Variables at *p* < 0.15 in univariable analysis were included and sequentially removed based on the Likelihood Ratio test. A *p*‐value of less than 0.05 was considered statistically significant.

## 3 Results

### 3.1 Study Population and Tumor Characteristics

During the 2018–2024 study period, 143 patients underwent curative‐intent robot‐assisted resection for histologically confirmed TET with detailed clinical and histologic data shown in Table 1. In total, there were 130 (90.9%) patients with thymoma and 13 (9.1%) patients with thymic carcinoma. The most common concomitant diseases were myasthenia gravis (*n* = 28, 19.6%) and hematologic disorders. Of all patients, 83.9% (*n* = 120) presented in pTNM Stage I and 16.1% (*n* = 23) were in pTNM Stage II or higher. Over the study period, surgical case volume increased and a higher proportion of patients with advanced tumor stage underwent RATS (Supporting Information S1: Figure S1). Of note, 7 patients were upgraded to TNM Stage IV at final pathology due to either lymph node involvement (*n* = 3, 2.1%), pleural metastasis (*n* = 2, 1.4%) or lung metastasis (*n* = 2, 1.4%).

**Table 1:** Demographic data and tumor characteristics.

Age at operation, years	63 (55–71)
Gender	
Male	64 (44.8%)
Female	79 (55.2%)
BMI	26.4 (23.3–29.3)
Smoking History	
Never	75 (52.4%)
Former	49 (34.3%)
Current	19 (13.3%)
Concomitant with autoimmune disease	
Myasthenia gravis	28 (19.6%)
Good's Syndrome	2 (1.4%)
PRCA	2 (1.4%)
PWCA	1 (0.7%)
Hashimoto's Thyroiditis	2 (1.4%)
Other	3 (2.1%)
Tumor size, mm	54 (35.5–75.0)
Subtype	
Type A thymoma	18 (12.6%)
Type AB thymoma	42 (29.4%)
Type B thymoma	62 (43.4%)
MTWLS	7 (4.9%)
Metaplastic thymoma	1 (0.7%)
Thymic carcinoma	13 (9.1%)
Masaoka‐Koga stage	
I	63 (44.1%)
II	57 (39.9%)
III	16 (11.2%)
IV	7 (4.9%)
pTNM stage	
I	120 (83.9%)
II	7 (4.9%)
III	9 (6.3%)
IV	7 (4.9%)
pT stage	
0^a^	1 (0.7%)
1	124 (86.7%)
2	7 (4.9%)
3	10 (7.0%)
4	1 (0.7%)
pN stage	
0	140 (97.9%)
1	2 (1.4%)
2	1 (0.7%)
pM stage	
0	139 (97.2%)
1a	2 (1.4%)
1b	2 (1.4%)
Neoadjuvant therapy	
Chemotherapy	7 (4.9%)
Chemoradiotherapy	1 (0.7%)
Adjuvant therapy	
Radiotherapy	19 (13.3%)
Chemotherapy	1 (0.7%)

*Note:* Demographic data and tumor characteristics. Values are given as median (interquartile range) or absolutes (%).

Abbreviations: MTWLS, micronodular thymoma with lymphoid struma; PRCA, pure red cell aplasia; PWCA, pure white cell aplasia.

a After neoadjuvant chemotherapy.

### 3.2 Perioperative Outcomes

**Figure 2:**
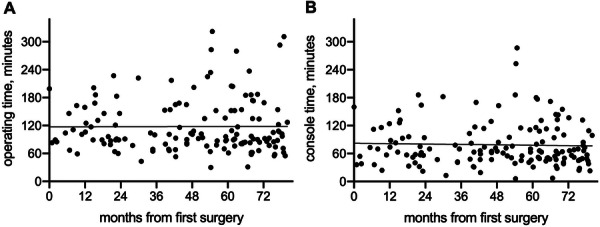
Duration of surgery. Total operating (A) and console (B) times are given according to time from the first RATS thymic tumor resection. No significant trend in operating times was observed.

Detailed information on the perioperative results can be found in Table 2. The median operation time was 96.5 min (IQR 81–152) with a median console time of 65 min (IQR 47–101.5). The estimated times for the duration of surgery did not decrease as the number of cases increased (Figure 2). The conversion rate to open surgery was 4.2% and all conversions were performed in a controlled environment to ensure optimal control of critical structures, there were no emergency conversions due to bleeding. R0 resection was achieved in 134 (93.7%) patients. Combined additional resections were performed in 44 (30.8%) patients, including pulmonary resections as wedge (*n* = 27, 18.9%) or lobectomy (*n* = 3, 2.1%) as well as resections of pericardium (*n* = 37, 25.9%) and major vessels (*n* = 4, 2.8%). Vessel resections included resection of the innominate vein in three patients and resection of the superior vena cava in one patient, which prompted conversion to hemiclamshell and was reconstructed using a polytetrafluoroethylene (PTFE) graft. Patients undergoing combined additional resections presented with higher tumor stage (Supporting Information S1: Figure S2) and the R0 resection rate was 84.1% in this subgroup; in contrast to 98.0% for patients without combined additional resections.

**Table 2:** Peritoperative outcomes.

Side of approach	
Right	28 (19.6%)
Left	115 (80.4%)
Conversion, total	6 (4.2%)
Thoracotomy	2 (1.4%)
Sternotomy	3 (2.1%)
Hemiclamshell	1 (0.7%)
Surgical margins	
R0	134 (93.7%)
R1/Rx	8 (5.6%)
R2	1 (0.7%)
Operating time, min	96.5 (81–152)
Console time, min	65 (47–101.5)
Combined additional resections,	
Number of involved structures	
0	99 (69.2%)
1	21 (14.7%)
2	19 (13.3%)
3	4 (2.8%)
Combined additional resections, structure	
Lung (wedge)	27 (18.9%)
Lung (lobectomy)	3 (2.1%)
Pericardium	37 (25.9%)
Major vessel	4 (2.8%)
Phrenic nerve resection	
No	137 (95.8%)
Yes	6 (4.2%)
Duration of ICU stay	
0 days	70 (49.0%)
1 day	62 (43.4%)
≥ 2 days	11 (7.7%)
Duration of chest tube, days	2 (1–3)
Duration of postop stay, days	4 (3–6)
Clavien‐Dindo complication grade^a^	
Grade 0	115 (80.4%)
Grade I	12 (8.4%)
Grade II	9 (6.3%)
Grade IIIa	2 (1.4%)
Grade IIIb	0 (0.0%)
Grade IV	5 (3.5%)
Grade V	0 (0.0%)
30‐days mortality	0 (0.0%)

*Note:* Perioperative outcomes. Values are given as median (interquartile range) or absolutes (%). Any deviation from the normal postoperative course without the need for pharmacological treatment or surgical, endoscopic, and radiological interventions; allowed regimens such as antiemetics, antipyretics, analgesics, diuretics, electrolytes, and physiotherapy; II: Requiring pharmacological treatment with drugs other than such allowed for grade I complications; blood transfusions and total parenteral nutrition are also included; III: Requiring surgical, endoscopic or radiological intervention under local anesthesia (IIIa) or general anesthesia (IIIb); IV: Life‐threatening complication requiring intermediate care/intensive care unit management; V: Death of a patient.

a Clavien‐Dindo Grade I.

Postoperatively, 73 (51.0%) patients were monitored for at least one night in the intensive care unit (ICU). The median duration of chest tube drainage was 2 days (IQR 1–3) and the median postoperative stay was 4 days (IQR 3–6). Postoperative complications occurred in 28 (19.6%) patients, of which 7 (4.9%) were classified as Clavien‐Dindo Grade III or higher serious complications. Combined additional resections were the only independent predictor of postoperative complications in a multivariable model including several patient and tumor characteristics (OR 2.873, *p* = 0.033; Table 3). Advanced TNM and Masaoka‐Koga stage were significant predictors in the univariable analysis but not in the multivariable model (OR 5.823, *p* = 0.002 and OR 3.825, *p* = 0.008, respectively). Of note, concomitant autoimmune disease did not significantly increase the risk of complications, nor did large tumor size ≥ 50 mm. There was no 30‐day mortality. The details of postoperative complications are reported in Supporting Information S1: Table S1.

**Table 3:** Univariable and multivariable logistic regression of risk factors for postoperative complications.

	Univariable			Multivariable	
Variables	OR (95% CI)	*p* value		OR (95% CI)	*p* value
Age ≥ 65	1.277 (0.565–2.890)	0.557			
Gender, male	1.004 (0.442–2.278)	0.993			
History of smoking	0.731 (0.320–1.669)	0.457			
BMI ≥ 30	0.883 (0.325–2.398)	0.807			
Concomitant with autoimmune disease	0.534 (0.187–1.520)	0.204			
Neoadjuvant treatment	2.515 (0.565–11.205)	0.226			
Tumor size ≥ 50 mm	1.580 (0.685–3.642)	0.283			
TNM Stage ≥ III	5.823 (1.906–17.794)	0.002		2.942 (0.833–10.389)	0.094
Masaoka‐Koga Stage ≥ III	3.825 (1.424–10.276)	0.008			
Conversion	8.960 (1.554–51.658)	0.014		4.828 (0.736–31.645)	0.101
R1/2 surgical margin	2.037 (0.354–11.708)	0.425			
Combined additional resections	4.565 (1.940–10.743)	< 0.001		2.873 (1.089–7.583)	0.033

*Note:* Risk factors for postoperative complications. Variables for the multivariable model were selected by backward stepwise selection based on the likelihood ratio test, considering variables with *p* values < 0.15 in the univariable analysis.

### 3.3 RATS Following Neoadjuvant Therapy

The study cohort included 8 patients who underwent RATS after neoadjuvant chemotherapy or chemoradiotherapy for tumors that were initially considered unlikely to be resectable (Supporting Information S1: Table S2). RATS was performed after reassessment of resectability and decision by an interdisciplinary board. Combined additional resections were required in all patients. In two patients, RATS was converted to open surgery to ensure optimal safety in the context of local infiltration of major vessels. R0 resection was achieved in four patients (50%). Patients with incomplete resection were managed as follows: one patient with R2 resection underwent secondary surgery under cardiopulmonary bypass, achieving R0 resection by extended resections of major vessels. Two patients with R1 resection proceeded to adjuvant radiotherapy, and one patient with R1 resection was not fit for further treatment due to a severe myasthenic crisis.

## 4 Discussion

In this study, we report a large, single‐center experience with RATS for thymic epithelial tumors. Our findings expand the scope of the robotic approach and provide important insights on current challenges for a broader application of this novel method. Overall, our results demonstrate the feasibility and safety of RATS for operable TETs, including large and locally advanced disease.

In locally advanced disease with infiltration of adjacent structures, complete resection often requires combined resections including lung resections, pericardial or large central vessel resections [4]. Recently, some authors have reported initial experience with minimally invasive procedures in locally advanced TET, demonstrating a broader use of RATS in selected patients with advanced disease [7, 16]. Similar to Huang et al., we demonstrated that combined additional resections of adjacent anatomic structures were an independent predictor of postoperative complications in a multivariable logistic regression model. This highlights the need for comprehensive postoperative surveillance [7]. The R0 resection rate in the group of patients who underwent combined additional resections of infiltrated anatomic structures was 84.1% compared to 98.0% in the patients with no infiltration of adjacent structures. Previous studies have not found a correlation between surgical approach and the likelihood of R0 resection, suggesting that complete resection is primarily influenced by the extent of local infiltration, rather than surgical technique [16, 17]. Overall, the R0 resection rate of 93.7% is similar to other large case series [7, 18, 19, 20]. The oncological equivalence of minimally invasive approaches to open surgery for the resection of TET was previously demonstrated both for VATS [6, 21] and for RATS [15, 18, 19, 20], and was not the subject of this study. It should be noted that objective criteria for resectability in locally advanced TET are difficult to define and preoperative imaging often remains unclear. Recently, we reported a high sensitivity of Cine‐MRI in predicting tumor infiltration of the great vessels, pointing a way to a more precise assessment of operability [14].

In fact, vascular involvement represents a common technical challenge during the minimally invasive resection of TET. In cases involving the innominate vein or its tributaries, segmental venous resection can be safely performed robotically using an endoscopic stapler, provided that proximal and distal vessel control is achieved [15, 22]. Minimally‐invasive resection of the superior vena cava with primary repair or patch plasty has been previously reported in selected patients [22], but is generally considered a reason for planned conversion in our practice [15, 23]. Conversely, pericardial resection is more frequently required during thymectomy when the tumor shows adherence to or direct invasion of the pericardium [24]. In most cases, limited pericardial involvement can be managed robotically through sharp dissection under high‐definition three‐dimensional visualization, thereby preserving cardiac integrity. The indication for reconstruction depends on both the size and the location of the defect [25]. The robotic system allows for precise intracorporeal suturing and patch tailoring, supporting the use of RATS in affected patients for both pericardial resection and, when indicated, reconstruction.

Some surgeons have suggested a maximum possible tumor size of a thymic tumor for surgical RATS resection of 5 to 8 cm [21, 26]. Recently, RATS resection of larger thymomas in size has also been reported [7, 18, 20]. Here, our findings highlight that tumor size ≥ 50 mm is not associated with increased postoperative complications. This is consistent with a previous study published by us, which showed comparable safety and oncological outcome after both RATS and open surgery of thymic tumors up to 50 mm in diameter [15]. Notably, the presence of concomitant autoimmune disease did not affect the likelihood of complications in line with previous evidence [27]. The conversion rate of 4.2% should be interpreted within a continuing expansion of indications for RATS including more complex cases, inherently encountering and pushing limits of RATS for intraoperative safety and control. Induction therapy has been suggested to improve resectability and survival in locally advanced disease deemed unresectable in initial surgical evaluation [28]. Neoadjuvant chemotherapy treatment regimens consist mostly of platinum and anthracycline‐based chemotherapy, and the combinations used for thymoma and thymic carcinoma are similar [29]. Patient selection in the context of induction strategies underlies a strong selection bias that is difficult to control, therefore estimating the efficacy and survival benefit of neoadjuvant therapy has proven difficult [28]. The overall response rates varied considerably in different studies, ranging between 4%–93% [29, 30]. The use of RATS in this context is largely unexplored. It should be noted that the eight patients we treated within a sequential therapy concept of RATS after induction therapy all presented with far advanced tumors and borderline resectability. We report an R0 resection rate of 50% in this cohort, and our assessment is that open procedures would likely not have allowed complete resection in the remaining patients due to extensive local infiltration. Given the small number of patients in this subgroup, the corresponding findings should be regarded as hypothesis‐generating and primarily descriptive, reflecting case‐report–level evidence. These data do not allow conclusions to be drawn about the efficacy of RATS after induction therapy, and further studies are needed to clarify the potential of RATS to achieve R0 resection with tolerable safety before robot‐assisted procedures can be readily endorsed as part of a neoadjuvant strategy. However, we believe that the main challenge lies not in the surgical approach, but in the reliable assessment of resectability in preoperative imaging.

We previously shared our initial experience with the learning curve for robotic surgeons during the implementation of a robotic program [31]. Although operating times decreased with growing case numbers for pulmonary lobectomy and simple thymectomy, this was not the case for mediastinal tumor resections [31]. Here, this observation is reiterated, again showing no significant reduction in console time or total operating time with growing case numbers. In our opinion, this is due to the continuing expansion of indications for RATS, leading to the inclusion of more complex cases over time.

This study is limited by its retrospective, single‐center design, which may lead to selection bias. In addition, generalizability and comparison with other centers may be limited due to individual clinical practices within a single center. Many patients have been treated recently and long‐term follow‐up is not yet available, and the significance of our results lies in perioperative outcomes and clinical findings rather than survival data. Furthermore, the relatively low number of postoperative complications may increase the risk of overfitting in the logistic regression model, and validation in larger cohorts is warranted. Given the rarity of TET, robust clinical trials are difficult to conduct and collaborative efforts such as the International Thymic Malignancy Interest Group (ITMIG) should be encouraged to facilitate multi‐center collaboration.

## 5 Conclusions

Robot‐assisted resection of TET is feasible and can be performed with satisfactory perioperative safety. Combined additional resections, which are often required for locally advanced disease, are an important predictor of postoperative complications. Given the challenges posed by large, locally advanced or pretreated TET, RATS requires adequate training and experience of the robotic surgeon and the entire surgical team and should be performed in high‐volume centers. Robot‐assisted resection of thymic epithelial tumors is feasible and can be performed with satisfactory perioperative safety.

## Author Contributions

**Benedikt Niedermaier:** conceptualization, formal analysis, investigation, writing – original draft, writing ‐ review and editing. **Nabil Khan:** investigation, writing – review and editing. **Florian Eichhorn:** investigation, writing – review and editing. **Maria Zehentmeier:** investigation, writing – review and editing. **Heidrun Grosch:** writing – review and editing. **Raffaella Griffo:** investigation, writing – review and editing. **Alessio Campisi:** writing – review and editing. **Antonia Margineanu:** writing – review and editing. **Michael Allgäuer:** investigation, writing – review and editing. **Petros Christopoulos:** writing – review and editing. **Michael Thomas:** writing – review and editing. **Hauke Winter:** writing – review and editing, supervision. **Martin E. Eichhorn:** conceptualization, supervision, investigation, writing – review and editing.

## Synopsis

- Robot‐assisted thoracoscopic surgery (RATS) for thymic epithelial tumors demonstrated high feasibility, with R0 resection achieved in 93.7% of patients and a low conversion rate to open surgery (4.2%).
- Postoperative complications occurred in 19.6% of patients, with combined extended resections emerging as the only independent predictor of adverse outcomes.
- These findings support the safety of RATS while highlighting increased risk in locally advanced cases requiring complex resections.

## Supporting information

Figure S1: Case volume per year according to Masaoka‐Koga stage. Figure S2: Masaoka‐Koga stage of patients undergoing combined additional resections.

## Data Availability

The data underlying this article will be shared on reasonable request to the corresponding author.
