# Supplementary material for: Impact of Combined Additional Resections on the Surgical Outcomes of Robot‐Assisted Resection of Thymic Epithelial Tumors
Source: J Surg Oncol. 2025 Dec 18;133(3):382–8. doi: 10.1002/jso.70162 (PMC12989189; doi:10.1002/jso.70162)
Supplement: Supplementary file 1 — Figure S1: Case volume per year according to Masaoka‐Koga stage. Figure S2: Masaoka‐Koga stage of patients undergoing combined additional resections. [file JSO-133-382-s001.docx]

Supplementary Material to:

**Impact of Combined Additional Resections on the Surgical Outcomes of Robot-Assisted Resection of Thymic Epithelial Tumors**

**Supplementary Figure S1:**


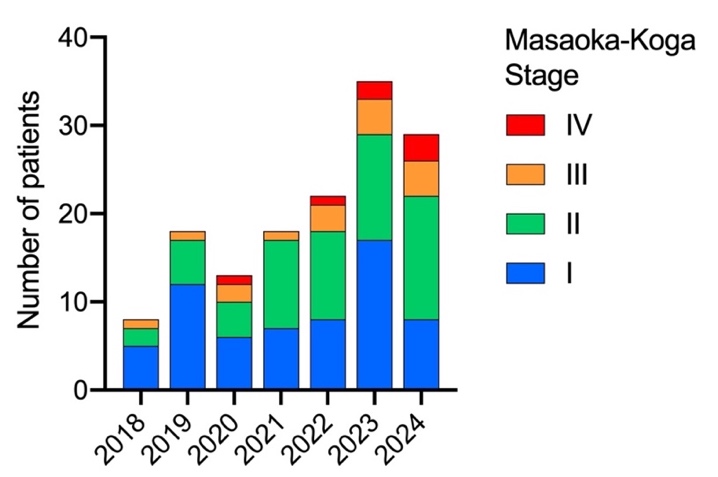


Figure S1: Case volume per year according to Masaoka-Koga stage.

**Supplementary Figure S2:**

*
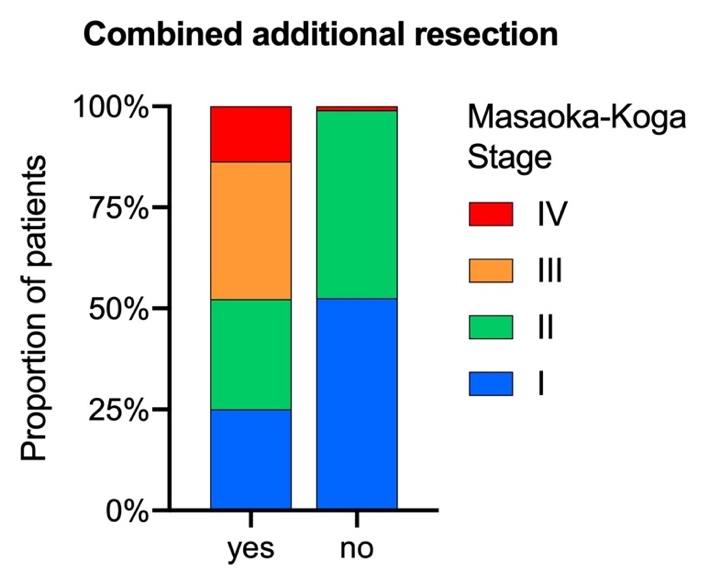
*Figure S2: Masaoka-Koga stage of patients undergoing combined additional resections.

**Supplementary Table S1:**

| Postoperative Complications | | |
| --- | --- | --- |
| Complications | **Number of Patients** | **Percentage** |
| Phrenic nerve injury | 7 | 4.9% |
| Pneumonia requiring antibiotics | 5 | 3.5% |
| Pleural effusion, not requiring intervention | 4 | 2.8% |
| Pleural effusion, requiring intervention | 1 | 0.7% |
| Myasthenic exacerbation | 2 | 1.4% |
| Acute respiratory distress syndrome | 2 | 1.4% |
| Pyothorax and sepsis, requiring intervention | 1 | 0.7% |
| Chylothorax, not requiring intervention | 1 | 0.7% |
| Pulmonary embolism | 1 | 0.7% |
| Atrial fibrillation | 1 | 0.7% |
| Positioning-related injury | 1 | 0.7% |
| Urinary tract infection | 1 | 0.7% |
| Leukopenia | 1 | 0.7% |

**Supplementary Table S2:**

| Robot-assisted resection of TET following neoadjuvant treatment | | | | | | | | | |
| --- | --- | --- | --- | --- | --- | --- | --- | --- | --- |
| ID | **Type** | **Initial tumor**  **size (mm)** | **Neoadjuvant**  **treatment** | **Pathologic**  **tumor size**  **(mm)** | **pStage** | **pM site** | **R** | **Combined**  **additional resection** | **Conversion** |
| 75 | B2 Thymoma | 130 | PAC | 146* | ypT1a, ypN0 | - | R0 | lung (wedge), pericardium | - |
| 76 | Basaloid Carcinoma | 85 | PAC | 83 | ypT4, ypN2 | - | R1 | lung (wedge),  innominate vein,  pericardium | - |
| 111 | SCC | 69 | Carboplatin/  Paclitaxel | 35 | ypT1a, ypN0 | - | R0 | lung (wedge), pericardium | - |
| 119 | B3 Thymoma | 100 | CHOP** | 83 | ypT1, ypN0  ypM1a | Pleura | R1 | lung (wedge) | - |
| 125 | SCC | 61 | Carboplatin/  Paclitaxel | 55 | ypT3, ypN0 | - | R1 | lung (wedge),  pericardium | - |
| 136 | B3 Thymoma | 84 | PAC | 91 | ypT3, ypN0 | - | R2 | lung (wedge),  pericardium | Sternotomy |
| 141 | AB Thymoma | 120 | Cisplatin/  Etoposide  Radiation  (50 Gy) | 120 | ypT0, ypN0 | - | R0 | lung (lobe),  VCS, pericardium | Hemi-  clamshell |
| 143 | SCC | 32 | Carboplatin/  Paclitaxel | 37 | ypT3, ypN1,  ypM1b | Lung | R0 | lung (wedge), pericardium | - |

Individual patient data is given. *****Multilobulated, total size given. ******Tumor was initially classified as T-cell lymphoma based on biopsy and CHOP administered on this basis. SCC, squamous cell carcinoma; PAC, cyclophosphamide, doxorubicin, cisplatin; CHOP, cyclophosphamide, doxorubicin, vincristine and prednisolone; Gy, Gray; VCS, vena cava superior
